# PBPK modeling of ivermectin—Considerations for the purpose of developing alternative routes to optimize its safety profile

**DOI:** 10.1002/psp4.12950

**Published:** 2023-03-15

**Authors:** Karen Rowland Yeo, David Wesche

**Affiliations:** ^1^ Certara UK Limited (Simcyp Division) Sheffield UK; ^2^ Certara Inc Princeton New Jersey USA

## Abstract

Although single‐dose ivermectin has been widely used in mass‐drug administration programs for onchocerciasis and lymphatic filariasis for many years, ivermectin may have utility as an endectocide with mosquito‐lethal effects at dosages greater and longer than those used to treat helminths. The final physiologically‐based pharmacokinetic (PBPK) model for ivermectin described here was able to capture, with reasonable accuracy, observed plasma drug concentration‐time profiles and exposures of ivermectin after a single oral dose of the drug in healthy male (dose range 6–30 mg) and female subjects, in both fasted and fed states, in African patients with onchocerciasis (150 μg/kg) and in African children. The PBPK model can be used for further work on lactation, pediatric dosing (considering CYP3A4 and Pg‐p ontogenies), and pregnancy, especially if nonstandard doses will be used. The key findings of our study indicate that absorption of ivermectin may be highly dependent on bile micelle‐mediated solubility. The drug is highly lipophilic and permeable, and its plasma exposure appears to be associated with the body mass index of an individual. These are all factors that need to be considered when extrapolating to more complex oral formulations or alternative routes of administration. Administering lower doses over a longer period may attenuate the dependence on bile micelle‐mediated solubility. With relevant inputs, the verified PBPK model developed here could be used to simulate plasma exposures following administration of ivermectin by complex generics in development.

## INTRODUCTION

Ivermectin is a long established veterinary endectocide that was first approved for human use in 1987 due to its antiparasitic activity against onchocerciasis, a skin and eye disease caused by the parasitic worm *Onchocerca volvulus* (*O. volvulus*).^1^ The disease is transmitted to humans through the bite of a black fly (genus *Simulium*) and, once matured in the human body, the adult female worm (macrofilaria) produces larval worms (microfilariae) that distributes in the skin and eyes. Although the microfilaricidal effect of orally administered ivermectin typically results in a rapid decrease in skin microfilarial load, the microfilariae begin to reappear in the skin 3–6 months after treatment as ivermectin does not kill adult worms residing in subcutaneous nodules. Multiple doses of ivermectin have been reported to reduce microfilarial load.^2^ Currently, mass‐drug administration (MDA) of ivermectin, consisting of a single dose once a year, has been implemented for this neglected tropical disease. Although biannual treatment with ivermectin has been considered for some disease foci, the additional cost and resource constraints are serious barriers to widespread implementation of this strategy.

The clinical pharmacology of orally administered ivermectin has been studied in healthy volunteers and in patients with Onchocerciasis. Safety and pharmacokinetics (PKs) of ivermectin at higher doses in healthy subjects were evaluated; following single doses of 30–120 mg, the area under the plasma concentration‐time curve (AUC) and maximum plasma concentration (*C*_max_) were generally dose proportional, with time to *C*_max_ (*T*_max_) ~4 h and terminal half‐life (*t*_1/2_) about 18 h.^3^ The geometric mean AUC of 30 mg ivermectin (range: 347–594 μg/kg) was 2.6 times higher when administered with food. Ivermectin was generally well‐tolerated, with no indication of associated central nervous system toxicity for doses up to 10 times the highest US Food and Drug Administration (FDA)‐approved dose of 200 μg/kg. In healthy and onchocerciasis volunteers treated with 150 μg/kg, no significant differences were found in the PK parameters.^4^

More recently, a clinical study was conducted to assess a new 18 mg ivermectin tablet in a fixed‐dose strategy of 18 and 36 mg single dose regimens, compared to the standard, weight‐based 150–200 μg/kg regimen.^5^ PK parameters showed a half‐life between 81 and 91 h in the different treatment groups. Body mass index (BMI) and weight were associated with *t*_1/2_ and total apparent clearance, which was attributed to the high lipophilicity of ivermectin, with longer retention times proportional to the presence of more adipose tissue. In the framework of a randomized controlled dose‐finding trial in rural Cote d'Ivoire, *Trichuris trichiura‐*infected pre‐school‐aged children (PSAC; 2–5 years old) and school‐aged children (SAC; 6–12 years old) were assigned to receive treatment with 100 or 200 μg/kg and 200, 400, or 600 μg/kg ivermectin, respectively.^6^ Both *C*_max_ and AUC_(0,72)_ increased in PSAC and SAC with ascending doses and were similar in both age groups when the current standard dose (200 μg/kg) was administered. PSAC with lower BMI were associated with significantly higher AUCs.

One potential additional benefit of ivermectin is that it has been shown to reduce the sporogony of *Plasmodium* in the mosquito and reduce transmission to humans.^7^ In 2016, the World Health Organization (WHO) held a technical consultation on ivermectin and subsequently published preferred product characteristics (PPCs) for endectocides against malaria using ivermectin as a reference product.^8^, ^9^ One of the key factors that was discussed was the optimal dosage/regimen required for transmission reduction with ivermectin MDA. The findings of a recent study indicated that adverse events following single‐dose treatment with up to 800 μg/kg of ivermectin occur without significant differences of frequency or intensity to those at regular approved doses.^10^ Currently, two potential regimens involving multiple higher oral doses are being investigated in malaria clinical trials. However, the efficacy of ivermectin is limited by the short‐lived effect of the drug in plasma, and long‐lasting formulations of ivermectin could offer a significant improvement over those currently available. Conducting appropriate clinical studies to investigate these complex generics is likely to be time‐consuming and costly. Modeling and simulation approaches could facilitate the selection of the most promising formulations for MDA early in the development process. One such mechanistic approach, physiologically based pharmacokinetic (PBPK) modeling, combines information on human physiology, demographics, and pharmacogenetics together with information on drug properties (physicochemical, binding, permeability, metabolism, and, if appropriate, transport) into a computational framework that allows plasma and tissue concentrations of a compound to be simulated.^11^ Predicted concentrations can then be used to compare against expected concentration‐related targets for efficacy and/or safety. Furthermore, PBPK models can be used to simulate exposures of molecules with complex absorption and disposition as well as investigate the effects of different formulations and routes of administration.^12^, ^13^, ^14^

A number of PBPK models with differing degrees of complexity and focus have already been developed for ivermectin.^15^, ^16^, ^17^ One PBPK model, which was able to describe the complex absorption and multiphasic distribution of ivermectin, was used to predict the PKs in patients with disseminated strongyloidiasis after a complex dosing regimen of multiple oral and subcutaneous dosing.^15^ The system parameters of the model were modified to account for the effects of the disease on physiology and hence on the PK of the drug. A second PBPK model, with more detailed disposition described by CYP3A4‐mediated metabolism and P‐gp‐mediated efflux, was used to determine optimal dosing regimens of ivermectin in adults and children for treatment of malaria.^16^ Finally, a minimal PBPK model with less well‐characterized metabolism and assuming first order absorption was used to simulate human lung exposure of ivermectin after oral administration.^17^ For a PBPK model to be adaptable for different routes of administration, it has to be able to describe adequately the absorption, distribution, metabolism, and excretion (ADME) of a drug so that it can be deconvoluted accordingly. It also has to be fully mechanistic so that it is applicable across populations and diseases, and system parameters can be modified accordingly to reflect associated changes in plasma protein binding, distribution, and metabolism. Furthermore, optimal in vitro ADME data should be used as inputs to the PBPK models. In a recent publication by Charman et al.,^18^ several methodological issues common to lipophilic compounds were identified, and customized experimental conditions were adopted for measurement of permeability, metabolism, and plasma protein binding of compounds, including ivermectin.

The initial objective of the work described here was to develop a fully mechanistic PBPK model for oral ivermectin using the updated in vitro data and verify the model in healthy adults and African patients with onchocerciasis and thereafter in children. The main objective was to identify key factors affecting the plasma exposure of ivermectin that are likely to require consideration when developing alternative routes of administration or complex formulations for MDA of ivermectin in an African population.

## METHODS

**FIGURE 1:**
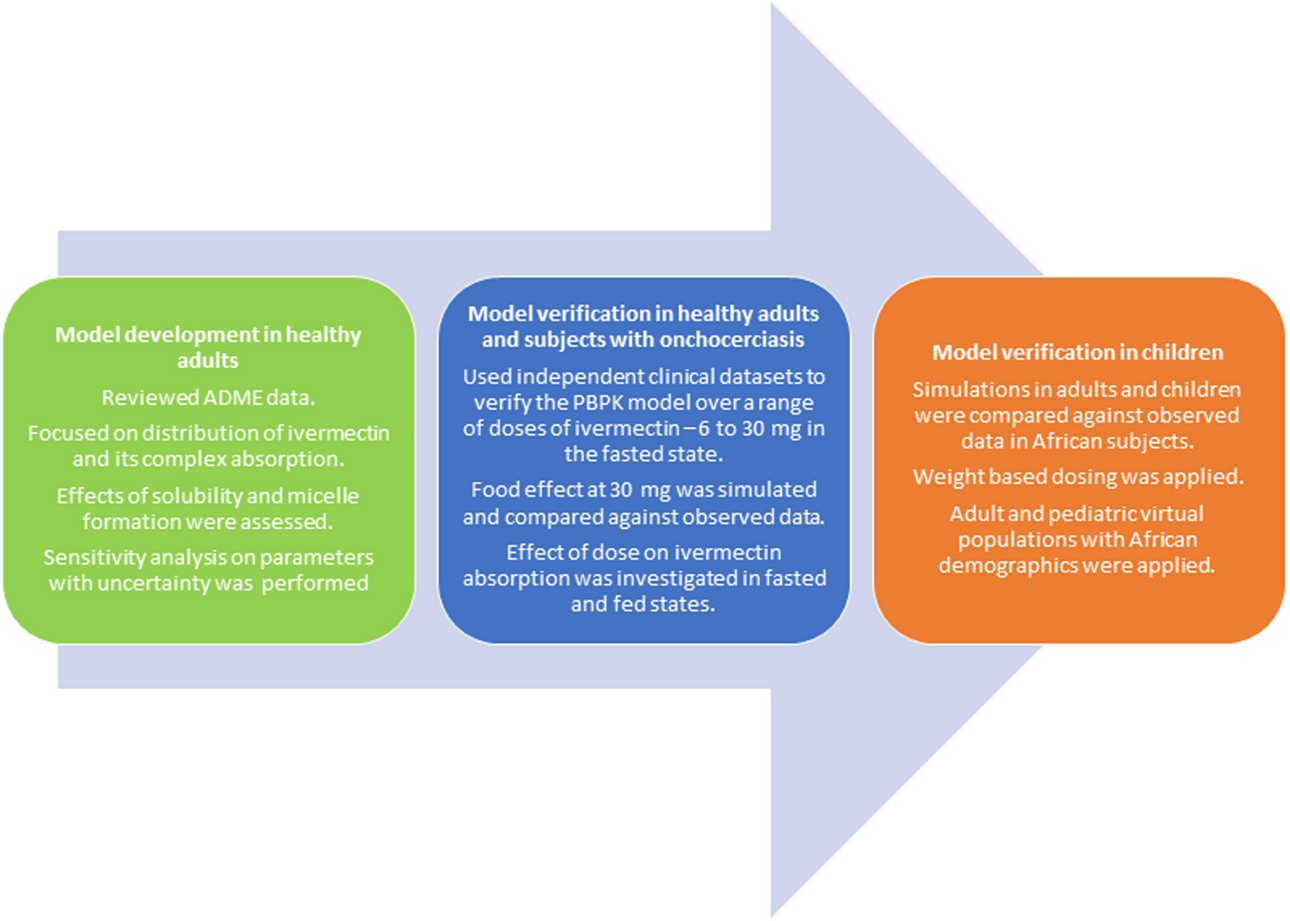
Workflow outlining the process used to develop and verify the PBPK model for ivermectin in healthy subjects, subjects with Onchocerciasis and children. ADME, absorption, distribution, metabolism, and excretion; PBPK, physiologically‐based pharmacokinetic.

The PBPK model for ivermectin was constructed in the Simcyp Simulator (version 21 release 1) using the workflow shown in Figure 1. The PBPK model was developed initially in healthy volunteers, then applied in African patients with onchocerciasis and African children. Ten simulated trials of virtual subjects with characteristics that matched (according to the number, age range, and proportion of women) those of the subjects used in each clinical study were generated. A base model was constructed for ivermectin using in vitro or in silico data (plasma protein binding, blood to plasma ratio, lipophilicity, rate of metabolism in human liver microsomes or human recombinant enzymes, and solubility). The performance of the final model was verified against independent clinical data not used in the model development or refinement.

### Model assumptions and input data

The PBPK model for ivermectin was developed using a full body PBPK model where different organs of the body are represented as compartments with specified blood flows, volumes, and tissue composition.^11^ The other organs were generally assumed to behave as well‐stirred compartments where distribution of the compound into the tissue is governed by standard perfusion rate‐limited PBPK model equations. Tissue:plasma partition ratios (Kp) were initially predicted using the approach outlined by Rodgers and Rowland and co‐workers.^19^, ^20^, ^21^ Absorption was described by the complex, regionally distributed permeability‐limited (advanced dissolution, absorption, and metabolism [ADAM]) model.^22^ As the mechanistic effective permeability model was used to predict regional permeability for ivermectin, passive transcellular permeability for unionized drug was estimated from the physicochemical data, mainly log P.^23^, ^24^ Initial input parameters for ivermectin (Table S1) and their derivation are discussed in detail in Appendix S1 and key parameters are mentioned briefly below.

#### Absorption

Ivermectin is classified as a Biopharmaceutics Classification System (BCS) Class II (high permeability and low solubility) compound. The solubility of ivermectin in simulated intestinal fluid with (FaSSIF) and without bile salts (SIF) has been measured at pH 6.5; values were 0.12 and 0.0007 mg/mL, respectively.^25^ Similar measurements were also performed to assess food effects on BCS class II compounds in general: the solubility of ivermectin in FaSSIF and FaSSIF blank (no surfactants) solutions was reported to be 0.0142 and 0.000175 mg/mL, respectively.^26^ It was noted that ivermectin strongly favored solubilization in micelles (98.8%). The effect of bile salts was to increase solubility by 171‐ and 81‐fold in the two independent reports. Thus, solubility was represented by aqueous phase solubility (governed by the Henderson−Hasselbalch equation for electrolytes at a given pH) and bile micelle‐mediated solubility.

Caco‐2 permeability experiments that were conducted previously for ivermectin were not able to generate apparent permeability coefficient estimates due to poor mass balance and inadequate sink conditions, frequently encountered with lipophilic compounds. Thus, the Caco‐2 permeability was determined as described by Katneni et al.^27^ using 10% plasma in the donor and acceptor chambers. Values of 190 × 10^−6^ cm/s and 62.1 × 10^−6^ cm/s were determined for ivermectin and propranolol (control), respectively. Efflux ratios of 1.5 and 97 were determined for ivermectin and rhodamine 123 (control), respectively.

#### Metabolism and transport

Ivermectin is extensively metabolized by human liver microsomes (HLMs) via hydroxylation and demethylation.^28^ In another study, 13 different metabolites (M1–M13) were identified after incubation of ivermectin with HLM.^29^ The three main metabolites (M1: 3″‐O‐demethyl‐ivermectin; M3: 4‐hydroxymethyl‐ivermectin; and M6: 3″‐O‐demethyl, 4‐hydroxymethyl‐ivermectin) were produced primarily by CYP3A4, and M1 was also produced to a small extent by CYP3A5. Although ivermectin has been identified as a P‐gp substrate^30^ and appears to undergo significant biliary excretion via this transporter,^31^ no in vitro kinetic data are available.

### Sensitivity analyses

**FIGURE 2:**
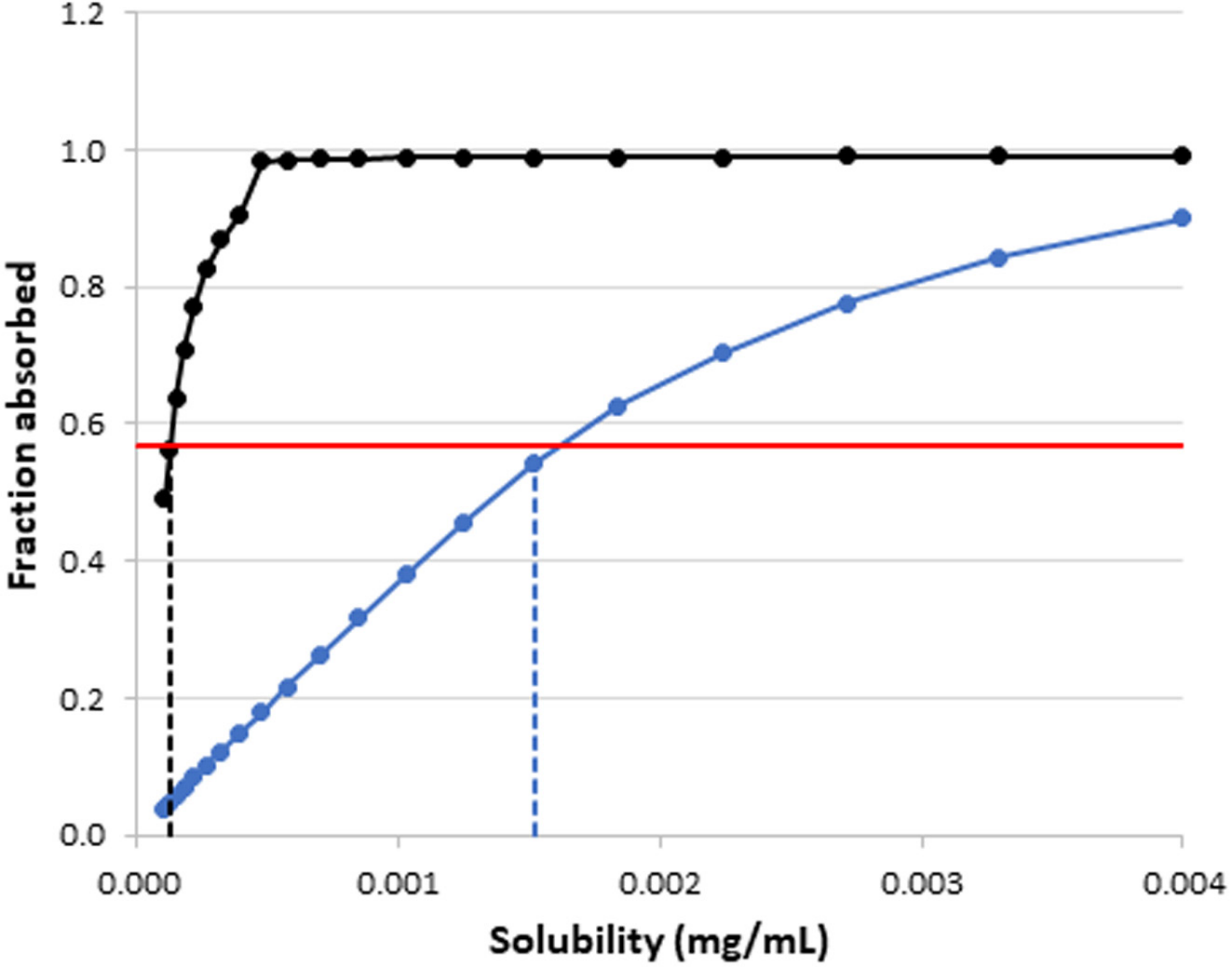
The effect of aqueous and total solubility (including bile‐micelle mediated solubility) on predicted *f*_a_ for ivermectin. Black line: total solubility, blue line: aqueous solubility, red line: indicates observed *f*_a_ of 0.57 from Edwards et al. (1998), dashed lines indicate solubilities that led to predicted *f*_a_ of 0.57. *f*_a_, fraction absorbed.

It is important to note that there is a significant degree of uncertainty and sensitivity associated with log P which is used to predict tissue Kp values, nonspecific microsomal binding in HLM and the bile‐micelle partition coefficient for the neutral species (Km:w,unionized) of ivermectin. Similarly, the aqueous solubility is another parameter of uncertainty and sensitivity. Thus, sensitivity of predicted PK parameters and plasma concentration profiles to a range of log P and aqueous solubility values was evaluated using local sensitivity analysis. The parameters of interest were adjusted over a range of values that spanned the initial input parameter range and the change in PK parameters or simulated output was evaluated. The results of the local sensitivity analysis involving solubility is shown in Figure 2 and the other analyses are shown in Appendix S1.

### Population characteristics

The healthy volunteer (HV) population was applied in initial simulations involving healthy subjects. With the exception of demographic data, all parameter values for the HV population are the same as those used for the North European Caucasian population (physiological parameters including liver volume and blood flows, enzyme abundances) details of which have been described previously.^32^ The clinical study in patients with onchocerciasis consisted of subjects living in South‐Eastern Nigeria, recruited from a community known to be hyperendemic for onchocerciasis.^33^ Thus, a virtual population based on a Black South African population that was published previously was applied.^34^ Details of this virtual population include data sources for age distribution, relationships between age, height and weight, and plasma protein levels. Simulations in African children were performed using the pediatric population, which includes extensive libraries on pediatric demography (age, height, weight, and body surface area), developmental physiology (liver size, renal function, and liver blood flow), and biochemistry (albumin and CYP ontogeny). The underlying algorithms describing these changes are reported elsewhere.^35^ As the pediatric population was based on a White population, the WHO growth charts from children (aged 2–11 years in West Africa) and weight‐for‐age growth curves based on pre‐existing population‐based anthropometric data, including individuals older than 14 days and younger than 50 years, from malaria‐endemic African countries were used to establish demographic profiles for the respective age groups.^36^ Details can be found in Appendix S1.

## RESULTS

### Effect of solubility and dose on the fraction absorbed

**FIGURE 3:**
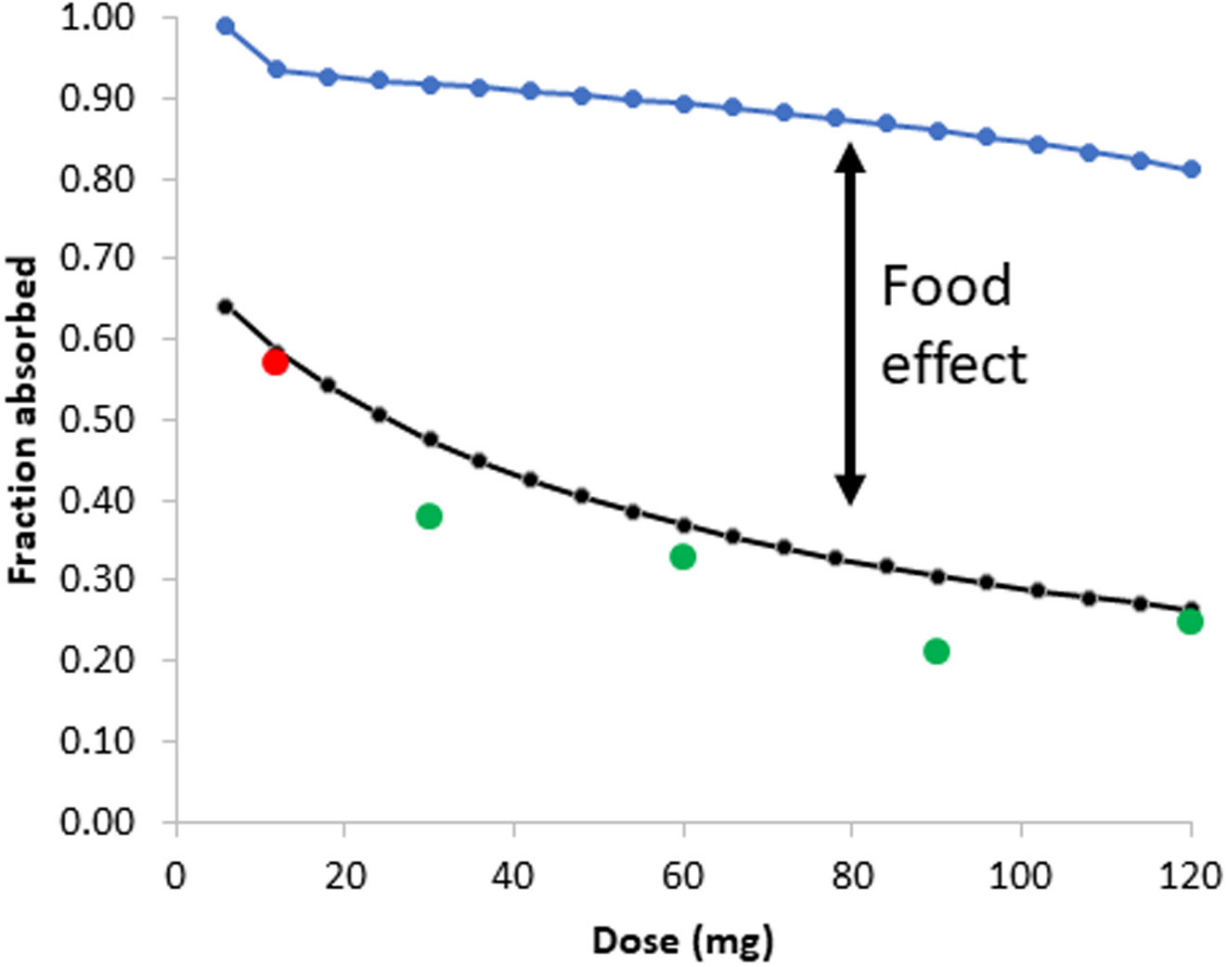
The effect of dose on the predicted *f*_a_ for ivermectin in the absence and presence of food intake. Black line: fasted state, blue line: fed state, red circle: clinical data from Edwards et al.,^37^ green circles: clinical data from Guzzo et al.^3^ *f*_a_, fraction absorbed.

The development of the ivermectin PBPK model is described in detail in Appendix S1. A full PBPK model in combination with the ADAM module was applied in simulations of plasma concentrations of ivermectin following oral administration of the drug. As seen in Figure 3, in the fasted state, the predicted fraction absorbed (*f*_a_) values are reasonably consistent with the observed data over the dose range from 12 to 120 mg, with a notable two‐fold decrease in *f*_a_ over this range. An observed *f*_a_

value was extrapolated from ivermectin exposure following administration of 12 mg in tablet versus solution, assuming complete absorption in the latter case.^37^ At higher doses (>30 mg), observed *f*_a_ values in the fasted state were estimated from the clinical study data reported by Guzzo et al.^3^ As the exposures were approximately linear above this dose, the exposures were corrected for dose and expressed as a fraction relative to the dose‐corrected exposure in the presence of food (assuming complete absorption). In the presence of food, where the applied bile salt concentrations are higher, the sensitivity analysis indicates that more than 80% of the drug is absorbed at all doses. In summary, the *f*_a_ appears to be sensitive to both dose and changes in bile salt concentrations.

### Simulations of plasma concentration‐time profiles of ivermectin in healthy subjects and patients with onchocerciasis

**FIGURE 4:**
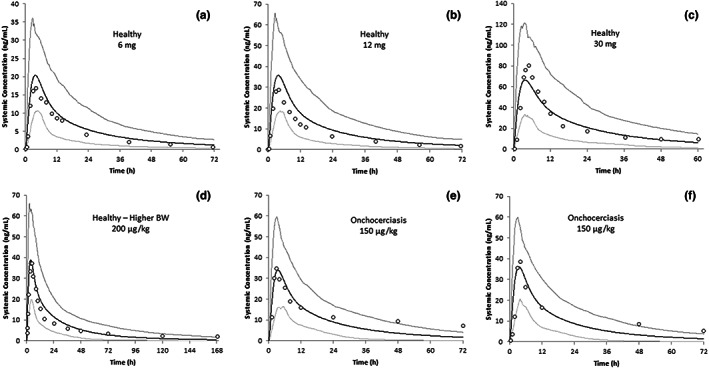
Simulated (lines) and observed (symbols) plasma concentrations of ivermectin in healthy subjects and subjects with onchocerciasis. Observed data are from NDA 50–742 for (a) and (b), Guzzo et al.^3^ for (c), Muñoz et al.^5^ for (d), Okonkwo et al.^33^ in (e) and (f) Elkassaby et al.^38^ BW, body weight.

Final input parameters for ivermectin that were used in all simulations described here are shown in Table S1. Simulated plasma concentrations of ivermectin in healthy male subjects following a single oral dose of 6, 12, or 15 mg ivermectin in the fasted state were reasonably consistent with observed data (Figure 4a,b, Table 1); *C*_max_, *T*_max_, AUC, and *t*_1/2_ values for ivermectin were within 1.31‐fold of observed data for all three doses. In a separate study involving a higher dose of 30 mg, simulated plasma concentrations of ivermectin in healthy subjects (48.5% female subjects) remained consistent with observed data (Figure 4c); *C*_max_, *T*_max_, AUC, and *t*_1/2_ values for ivermectin were within 1.23‐fold of observed data (Table 1). In addition, the predicted 2.2‐fold increase in exposure as a consequence of food was reasonably consistent with the observed value of 2.6‐fold.^3^ In both the fasted and fed states, the variability in exposure is high due to the variability in bile salt concentrations. The critical micelle concentrations must be attained for the bile‐micelle mediated solubility to be applied.

**TABLE 1:** Mean simulated and observed PK parameters for ivermectin in healthy adults and subjects with onchocerciasis following a single oral dose.

Clinical study design		*C*_max_	*T*_max_	AUC _(0,72)_	*t*_1/2_	Reference
		(ng/mL)	(h)	(ng h/mL)	(h)	
6 mg *n* = 12 Male	Simulated	22.1	4.3	385	29	
	Observed	18.3	3.9	347	n/a	NDA 50‐742
	Prediction error	1.21	1.11	1.11		
12 Mg *N* = 12 Male	Simulated	38.6	4.4	671	28	
	Observed	30.6	3.8	513	n/a	NDA 50‐742
	Prediction error	1.26	1.15	1.31		
15 mg *n* = 12 male	Simulated	41.0	3.9	673	33	
	Observed	48.5	3.8	820	n/a	NDA 50‐742
	Prediction error	0.85	1.02	0.82		
30 mg *n* = 12 50% female	Simulated	72.1	4.4	1596	24.7	Guzzo et al. (2002)
	Observed	84.8	4.6	1724	20.1	
	Prediction error	0.85	0.96	0.93	1.23	
200 μg/kg *n* = 57 60% female	Simulated	41.8	4.2	949	49.8	Muñoz et al. (2018)
	Observed	43.2	4.2	1088	80.7	
	Prediction error	0.97	1.00	0.87	0.62	
150 μg/kg *n* = 12 Nigerian 33% female	Simulated	41.1	4.1	781	28.4	Okonkwo et al. (1993)
	Observed	38.2	4.7	1545	56.5	
	Prediction error	1.08	0.87	0.51	0.50	
150 μg/kg *n* = 10 Sudanese male	Simulated	38.5	4.0	687	29.1	Elkassaby et al. (1991)
	Observed	38.0	5.6	1032	16.0	
	Prediction error	1.01	0.72	0.67	1.82	

Abbreviations: AUC, area under the plasma concentration‐time curve; *C*_max_, maximum plasma concentration; PK, pharmacokinetic; *t*½, terminal half‐life; *T*_max_, time to maximum plasma concentration.

In healthy subjects weighing between 50 and 109.9 kg (59.6% female subjects), predicted *C*_max_ and AUC for ivermectin were within 1.13‐fold of observed data (Table 1) and the *t*_1/2_ within 1.6‐fold following a single oral dose of 200 μg/kg ivermectin in the fasted state. In the clinical study, the higher BMI (adipose volume) overall led to a longer half‐life (due to the higher volume of distribution) than reported for other studies (Table 1). This trend was reflected in the simulated results. Predicted *C*_max_ and AUC for ivermectin were within 2.00‐fold of observed data for Nigerian patients (33% female patients)^30^ and within 1.50‐fold of observed data for Sudanese male patients^38^ with onchocerciasis following a single oral dose of 150 μg/kg ivermectin in the fasted state. In the former case, the *t*_1/2_ was underpredicted by two‐fold whereas in the latter case, it was overpredicted by two‐fold. Doses of 12–15 mg are equivalent to 150–200 μg/kg ivermectin. Whereas the *C*_max_ was consistent across the clinical studies, there were significant differences in the *t*_1/2_ which appeared to be largely dependent on BMI (adipose volume).

### Simulations of plasma concentration‐time profiles of ivermectin in African children

**FIGURE 5:**
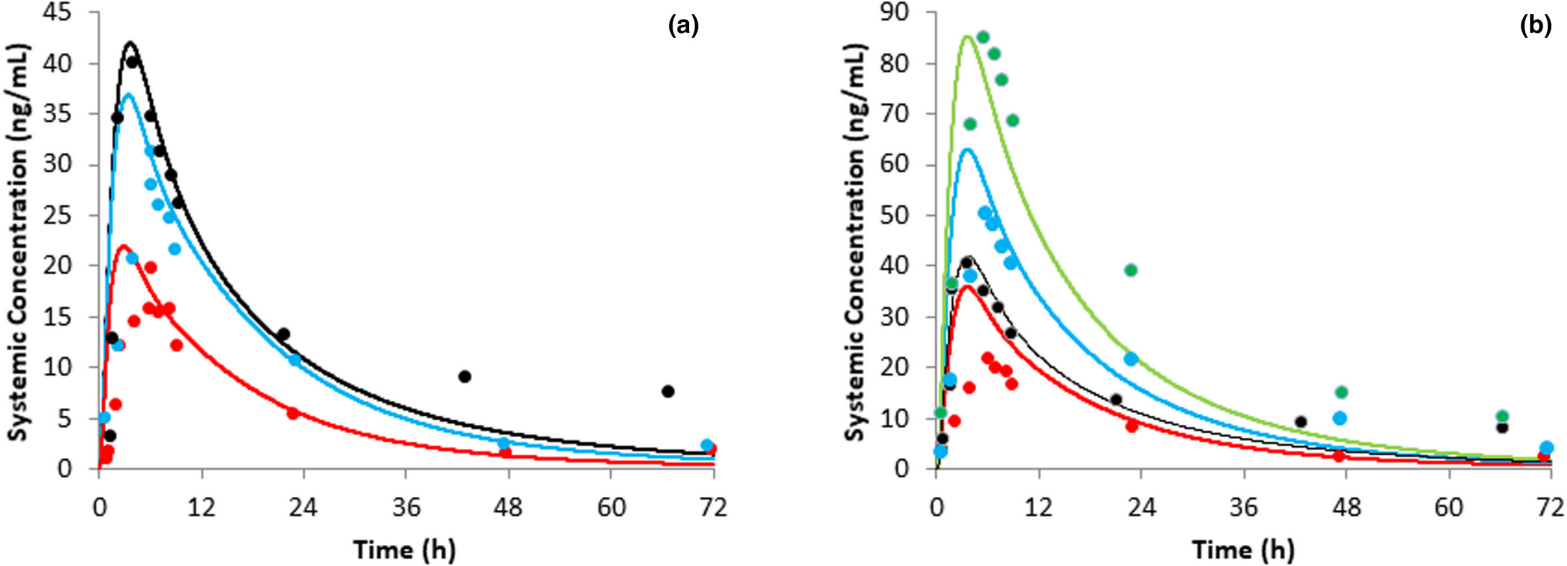
Simulated (lines) and observed (symbols; Schulz et al.,^6^) plasma concentrations of ivermectin in (a) adults (black – 200 μg/kg) and children aged 2–5 years (blue – 200 μg/kg; red – 100 μg/kg) and in (b) adults (black – 200 μg/kg) and children aged 6–12 years (red ‐ 200 μg/kg; blue – 400 μg/kg; green – 600 μg/kg) following a single oral dose of ivermectin in the fasted state.

Simulated and observed^6^ plasma concentrations of ivermectin in adults and PSAC (aged 2–5 years) following a single oral dose of 100 or 200 μg/kg ivermectin are shown in Figure 5a. Corresponding simulations for SAC (aged 6–12 years) following a single oral dose of 200, 400, or 600 μg/kg ivermectin are shown in Figure 5b. The adult data were determined in an independent study^39^ comparing samples obtained from plasma and dried blood spot (DBS): for each matrix, the detected *C*_max_ was 51.6 ng/mL (26.7–91.2 ng/mL) and 40.1 ng/mL (22.6–64.3 ng/mL), respectively. The reported AUC was 987 ng h/mL for plasma and 810 ng h/mL for DBS samples. Although no statistically significant difference was observed between the PK parameters for the two matrices (small sample size; *n* = 11), the *C*_max_ and AUC were 1.29‐ and 1.22‐fold higher, respectively for plasma than for DBS analysis. As the observed data in children were based on DBS as the matrix rather than plasma, a correction was applied to the observed data (*C*_max_ – 1.29; AUC – 1.22) in order to be able to directly compare against the simulated profiles and PKs. Thereafter, the simulated *C*_max_ and AUC values for ivermectin were within 1.31‐fold of observed data for the PSAC and within 1.33‐fold of observed data at the two lower doses and within 1.59‐fold at the higher dose for the SAC (Table 2). It should be noted that in both PSAC and SAC, *T*_max_ values were underpredicted. It is also important to recognize that 200 μg/kg in adults is equivalent to about 12 mg, whereas in children aged 2–5, it is on average 2.6 mg; predicted values of *f*_a_ were 0.52 and 0.71, respectively.

**TABLE 2:** Mean simulated and observed PK parameters for ivermectin in PSAC and SAC after a single oral dose of ivermectin in the fasted state.

		*C*_max_	*t*_max_	AUC_(o,∞)_	*t*_1/2_	AUC relative to adult
Group		(ng/mL)	(h)	(ng*h/mL)	(h)	
Adult	Simulated	44.0	3.9	756	21.9	1.00
200 μg/kg	Observed	51.6	3.9	987	26.2	1.00
*N* = 11	Prediction error	0.85	1.0	0.77	0.84	
PSAC	Simulated	41.1	4.3	705	25.7	0.93
200 μg/kg	Observed	31.5	5.9	610	16.3	0.62
*N* = 39	Prediction error	1.31	0.72	1.16	1.58	
PSAC	Simulated	22.9	3.5	367	16.4	0.49
100 μg/kg	Observed	20.0	6.0	378	17.3	0.38
*N* = 41	Prediction error	1.14	0.59	0.97	0.95	
SAC	Simulated	38.0	3.9	614	22.1	0.81
200 μg/kg	Observed	28.3	6.0	808	18.1	0.82
*N* = 41	Prediction error	1.35	0.66	0.76	1.22	
SAC	Simulated	64.7	3.9	1035	20.6	1.37
400 μg/kg	Observed	52.5	6.0	1288	18.1	1.31
*N* = 39	Prediction error	1.23	0.66	0.80	1.14	
SAC	Simulated	87.2	3.9	1420	19.9	1.88
600 μg/kg	Observed	85.3	6.0	2237	18.1	2.27
*N* = 40	Prediction error	1.02	0.66	0.63	1.10	

*Note*: Observed data are from Schulz et al.^6^

Abbreviations: AUC, area under the plasma concentration‐time curve; *C*_max_, maximum plasma concentration; PK, pharmacokinetic; PSAC, pre‐school‐aged children; SAC, school‐aged children; *t*½, terminal half‐life; *T*_max_, time to maximum plasma concentration.

## DISCUSSION

Ivermectin (Stromectol) tablets received FDA approval for the treatment of onchocerciasis and strongyloides more than 25 years ago and has been the mainstay of treatment for onchocerciasis elimination programs through MDA. Although the safety and PKs of ivermectin were assessed in a multiple‐dose clinical pharmacology study^3^ in HVs at doses ranging from 30 to 120 mg (300–2000 μg/kg), the vast majority of our confidence in safety comes from annual MDA of single 200 μg/kg doses to millions of people at risk.^40^ More recently, ivermectin has been proposed as an endectocide for malaria transmission control. The WHO developed a PPC document, which provides a useful framework of thinking about the prospective development of a drug that results in a blood concentration of parent and/or metabolites that are lethal to the mosquito, resulting in transmission reduction at the population level.^41^ In essence, there is no direct benefit of the drug to individuals, but a possible indirect benefit to individuals as members of a community in which they reside. Thus, confidence in safety for adults and special populations must clear a high hurdle. Preliminary studies to date have indicated that higher multiple dosages of ivermectin are necessary for a reduction in malaria transmission.^7^ There is a growing awareness of the PK/pharmacodynamic (PD) aspects of ivermectin's activity against the mosquito following a blood meal.^7^ The current PPC document calls for endectocides to be given once a month to provide at least 30 days of effective coverage.^37^ Data to date indicate that ivermectin is unlikely to provide effective coverage for 30 days. Rather than empiric dose/regimen selection, model‐informed drug development tools (empiric PK models and PBPK models) and principles should guide decisions and investments based on available information. Because higher doses are likely to be needed for a sustained effect, PBPK modeling can help inform the likelihood of success, whether drug accumulation is likely (both central compartment and tissues), impact on fetal concentrations (ivermectin is pregnancy category C – is teratogenic in animals [NDA 50–742]), impact on infants of lactating mothers, drug‐drug interactions (DDIs), etc. Furthermore, it could be used to define the exposure metric over time, and with the use of a parasite growth‐death *E*_max_ PD model, the impact of ivermectin on macrofilarial viability and female fecundity over time to assess whether transmission can be interrupted after multiple doses could be assessed.^42^, ^43^ PBPK modeling of ivermectin provides a framework of thinking around a plausible mechanism of PK‐PD interaction for this parasite.

The final PBPK model described herein was able to capture, with reasonable accuracy, observed plasma drug concentration‐time profiles and exposures of ivermectin after a single oral dose of the drug in healthy male (dose range 6–30 mg) and female subjects, in both fasted and fed states, and in African patients with onchocerciasis (150 μg/kg). The model was also used in simulations of pre‐school (PSAC: 100 and 200 μg/kg) and school aged (SAC: 200, 400, or 600 μg/kg) children; predicted ivermectin exposures (*C*_max_ and AUC) in PSAC were within 1.31‐fold of observed data and in SAC, within 1.33‐fold of observed data at the two lower doses and 1.59‐fold at the higher dose. A 200 μg/kg dose is equivalent to 12 to 15 mg in adults and about 3 mg in PSAC. Thus, the dose‐dependent change in fa needs to be accounted for in dose extrapolations (0.52 vs. 0.71). The final PBPK model for ivermectin was sensitive to changes in BMI and factors that affect the solubility of the drug in the gastrointestinal tract (bile salt concentrations increased by food intake). This is important because bile salt concentrations in malnourished populations may differ from White populations and are likely to contribute significantly to the variability associated with the plasma exposure of ivermectin. The PBPK model can also be used for further work in lactation, pediatric dosing (considering CYP3A4 and Pg‐p ontogenies),^44^ and pregnancy,^45^ especially if nonstandard doses will be used.

As the PBPK model presented here captures the in vivo ADME characteristics of ivermectin and allows deconvolution of the individual processes, it can be applied with confidence to describe the disposition of complex generics using relevant input parameters and/or modules describing other routes of administration (ROAs). Key findings of our study indicate that absorption of ivermectin may be highly dependent on bile micelle‐mediated solubility. The drug is highly lipophilic and permeable, and its plasma exposure appears to be associated with the BMI of an individual. These are key factors that need to be considered when extrapolating to more complex oral formulations or alternative ROAs. Slow‐release formulations of ivermectin, currently under study, are already demonstrating positive preliminary results on the efficacy and safety of implants. However, the scalability of implants in an MDA setting could be challenging. Thus, other formulations such as patches or innovative gastric retention devices are also being developed.^46^ Administering lower doses over longer periods may attenuate the dependence on bile micelle‐mediated solubility. Lyndra's investigational long‐acting oral biweekly ivermectin (LYN‐163) product, enabled by Lyndra's LYNX drug delivery platform, is being investigated in a phase I study currently, as a tool in the fight to eradicate malaria. The platform is able to deliver the drug consistently, minimizing peaks and troughs of drug levels compared to daily medicine. Moving away from oral formulations altogether would mitigate the solubility issues, especially at high doses and dependency on food/micelle interactions.

The high degree of lipophilicity/permeability may lend itself to dermal or intramuscular formulations. Indeed, MedinCell is developing a subcutaneous injection of a long‐acting formulation of ivermectin available in the form of a pre‐filled syringe, ready‐to‐use, with 24‐month stability at room temperature. MedinCell's BEPO technology will allow the formation of a small subcutaneous depot, fully bioresorbable, at the time of injection. It will act as a biodegradable mini pump that releases ivermectin regularly until it is completely bioresorbed. Although being developed for coronavirus disease (COVID), this long latency afferent inhibition could be considered for malaria intervention. However, it should be recognized that for MDA, the formulations must be cost‐effective.^47^

Ivermectin is a substrate of CYP3A4^24^ and P‐gp^26^ and appears to undergo significant biliary excretion via this transporter and potentially BCRP. A PBPK model accounting for CYP3A4‐mediated metabolism in the liver and P‐gp‐mediated efflux in the intestine was developed previously^48^ and used to assess the DDI liability of ivermectin with CYP3A4 perpetrators. As there are no clinical DDI studies with established CYP3A4 perpetrators, it is not possible to verify the contribution of CYP3A4 (fm_CYP3A4_) to the clearance of ivermectin (100%; Badhan et al.^17^). In the model presented here, the fm_CYP3A4_ is less than 8% and the majority of the clearance is assigned to biliary excretion. This is in line with the finding that accumulation of ivermectin (10‐fold) occurred in the gall bladder cells in P‐gp‐deficient mice relative to control mice.^48^ In addition, in 42 patients treated with ivermectin, a significant difference in MDR1 variant allele frequency was observed between suboptimal responders and responders.^49^ Furthermore, in the PBPK model published by Badhan et al.,^41^ there were no available in vitro data to describe the intestinal P‐gp‐mediated efflux of ivermectin. Thus, it was assumed that the active efflux was similar to that of digoxin and a sensitivity analysis was performed to demonstrate that these input parameters were able to capture the observed nonlinearity associated with *C*_max_ at the higher doses. In our PBPK model, the nonlinearity is explained by dose‐limited solubility enhanced by bile micelle formation. Indeed, because of the high passive permeability for ivermectin that was determined in a Caco‐2 system (Appendix S1), it was assumed that intestinal efflux is likely to be negligible compared to passive permeability. Although the performance of the two models is similar in terms of simulated exposures over the clinical dose range, predicted DDIs for ivermectin mediated by CYP3A4 perpetrators are significantly greater using the model reported by Badhan et al.^17^; a 70%–80% versus 10%–20% reduction in exposure was predicted during coadministration of the moderate CYP3A4 inducer efavirenz. As ivermectin is often co‐prescribed with other drugs, accurate assessment of the DDI liability of the drug is required. The fact that few clinically significant DDIs with ivermectin have been reported over the years, lends weight to the smaller contribution of CYP3A4 to the disposition of the drug assigned in our model. The negligible contribution of intestinal P‐gp‐mediated efflux and metabolism by CYP3A4 indicates that the DDI liability of ivermectin with CYP3A4/P‐gp perpetrators is likely to be similar irrespective of the ROA.

In summary, the verified PBPK model described here can be used for further work in lactation, pediatric dosing (considering CYP3A4 and Pg‐p ontogenies), pregnancy, especially if nonstandard oral doses will be used. With relevant inputs, the model could be used to simulate plasma exposures following administration of ivermectin by a range of complex generics. Furthermore, when appropriate ADME data become available for the main metabolites, the model could be expanded to include these moieties, especially if they are found to be active.

## FUNDING INFORMATION

The Bill & Melinda Gates Foundation funded the development of the ivermectin model.

## CONFLICT OF INTEREST STATEMENT

K.R.Y. and D.W. are employees of Certara UK Limited (Simcyp Division)/Certara. As an Associate Editor for *CPT: Pharmacometrics & Systems Pharmacology*, Karen Rowland Yeo was not involved in the review or decision‐making process for this paper.

## Supporting information


Appendix S1
