# Supplementary material for: PBPK modeling of ivermectin—Considerations for the purpose of developing alternative routes to optimize its safety profile
Source: CPT Pharmacometrics Syst Pharmacol. 2023 Mar 15;12(5):598–609. doi: 10.1002/psp4.12950 (PMC10196439; doi:10.1002/psp4.12950)
Supplement: Supplementary file 1 — Appendix S1 [file PSP4-12-598-s001.docx]

SUPPLEMENTARY INFORMATION

## Part A: Model assumptions and input data for ivermectin

### *Physicochemical data*

A molecular weight of 875.1 was used for ivermectin. The drug was assumed to be neutral at physiological pH. A range of log P values have been reported for ivermectin; a calculated value (cLog P) of 4.1 to a measured value > 7. In the latter experiment, due to the lipophilic nature of the compound, very low concentrations were expected in the aqueous buffer phase samples of the partition assay and therefore, a highly sensitive LCMS method was developed for analysis of ivermectin with detection limits of 0.1 ng/mL. The ivermectin partitioning experiment was conducted in octanol / aqueous buffer mixtures at pH 7.4 (50 mM phosphate buffered saline (PBS)). Despite a highly sensitive method and high incubation concentration (10 mg/mL), no measurable concentrations of the compound were detected in aqueous phase samples (concentrations conservatively < 1 ng/mL) while octanol sample concentrations were similar to the target concentration (10 mg/mL). The absence of detected compound in the aqueous phase of the partitioning experiments supports the highly lipophilic nature of the compound. While a Log D value could not be measured for ivermectin, it does appear to be high (estimated as >7 using a conservative analytical limit of quantitation of 1 ng/mL). However, the possibility of artefacts due to compound adsorption to the incubation tubes cannot be excluded. Given the uncertainty of the measured log P value, a predicted partition coefficient (cLogD7.4) of 5.83 (ChemAxon Instant JChem Version 18.5.0) was also assessed. Where relevant, sensitivity analysis was performed to investigate the effect of a range of log P values on predicted ADME parameters to be used in simulations.

### *Absorption*

While the absolute bioavailability of ivermectin has not been determined in humans, the use of a liquid formulation (12 mg ivermectin) resulted in a 1.57-fold increase in bioavailability compared to the tablet formulation (Edwards *et al*., 1988). Furthermore, the geometric mean AUC of 30 mg ivermectin was 2.6-fold higher when administered with food (Guzzo *et al*., 2002). Thus, it appears that assuming complete absorption from solution in the fasted state or tablet in the fed state, the fraction absorbed from a tablet in the fasted state is likely to range from 38 to 60%, depending on the dose.

Ivermectin is classified as a BCS Class II (high permeability, low solubility) compound. As the mechanistic P_eff_ model (Reynolds *et al.,* 2009; Sugano, 2009a; Sugano, 2009b) was used to predict regional permeability for ivermectin, passive transcellular permeability for unionised drug was estimated from the physicochemical data, mainly log P (Sugano, 2009a; Sugano, 2009b). A sensitivity analysis, conducted to assess the impact of log P on the regional permeability (P_eff,man_), indicated that above a log P value of 5.83, P_eff,man_ was relatively insensitive to any change (data not shown).

The aqueous solubility of ivermectin is reported to be 0.004 mg/mL (Takano *et al*., 2006). The solubility of ivermectin in simulated intestinal fluid with (FaSSIF) and without bile salts (SIF) has been measured at pH 6.5; values were 0.12 and 0.0007 mg/mL, respectively (Takano *et al*., 2006). Similar measurements were also performed to assess food effects on BCS class II compounds (Rahman, 2016): the solubility of ivermectin in FaSSIF and FaSSIF blank (no surfactants) solutions was reported to be 0.0142 and 0.000175 mg/mL, respectively. It was noted that ivermectin strongly favoured solublisation in micelles (98.8%). Thus, the effect of bile salts was to increase solubility by 171- and 81-fold in the two independent reports. Aqueous solubility measurements are devoid of the bile micelle-mediated solubility effect. Thus, a bile-micelle partition coefficient for the neutral species (Km:w,unionized) of ivermectin was estimated using a built-in linear regression equation based on log

P (Equation 1). At log P values of 4.1, 5.83 and 7, using an aqueous solubility of 0.000175 mg/mL, the total solubility increased by 12.4-fold, 218-fold and 1595-fold, respectively, when bile micelle-mediated solubility was considered. Thus, as part of the initial model development, the effect of solubility and log P on the fraction of ivermectin absorbed was assessed.

Equation 1 describes the total solubility (S_Tot_) which is a composite function of the aqueous phase solubility (governed by the Henderson−Hasselbalch equation for electrolytes at a given pH), bile micelle-mediated solubility of the drug and an excipient phase solubility (S_bound,excip_).


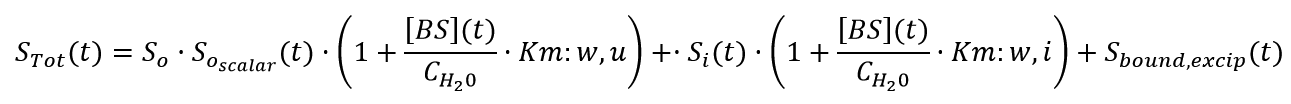
 (Equation 1)

[BS] is the concentration of bile salt (sodium taurocholate:lecithin molar ratio 4:1); S_0_ is the aqueous intrinsic solubility; S_i_ refers to the aqueous phase solubility of the ionized form of the drug at a given pH; C_H2O_ is the concentration of water (55.56 mM); and Km:w,u/i are the bile micelle:water partition coefficients for neutral (or ionized) molecular species, respectively. Initial estimates of Km:w values can be obtained using Equation 2 where a = 0.74 and b = 2.29 for sodium taurocholate/lecithin (4:1 ratio) mixtures.

$$logK_{m:w,unionised}=0.745\cdot logP_{o:w}+2.291$$

(Equation 2)

Caco-2 permeability experiments that were conducted previously for ivermectin were not able to generate P_app_ estimates due to poor mass balance and inadequate sink conditions, frequently encountered with lipophilic compounds (Takano *et al.,* 2006). Thus, the Caco-2 permeability was determined as described by Katneni *et al.* (2018) using 10% plasma in the donor and acceptor chambers. Permeability data for propranolol and rhodamine 123 compared well with in-house validation data confirming the functional integrity and activity of P-gp/MRP-mediated efflux in the Caco-2 test system. Control compounds showed very similar P_app_ values in 10% plasma compared to buffer. Caco-2 cell monolayer integrity and P-gp transporter function were unaffected by the presence of human plasma in the donor and acceptor chambers. Values of 190 x 10^-6^ cm/s and 62.1 x 10^-6^ cm/s were determined for ivermectin and propranolol (control), respectively. Efflux ratios of 1.5 and 97 were determined for ivermectin and rhodamine 123 (control), respectively.

### *Plasma protein binding and volume of distribution*

Plasma protein binding was determined *via* rapid equilibrium dialysis (RED) with incorporation of a presaturation step, based on methods reported previously (Charman *et al.,* 2020). Prior to using in the binding assay, RED inserts were exposed to each compound in order to saturate non-specific binding sites and thereby accelerate equilibration. Diluted human plasma (10% (v/v) in PBS) was spiked with ivermectin (at 2000 ng/mL) and dialysed against PBS containing a low concentration of the compound (100 ng/mL) for 24 h. As the binding assay was performed using diluted human plasma, data were corrected for the dilution factor to give a binding value for neat human plasma *via* an established approach which accounts for the shift in equilibria that occurs with protein dilution (Kalvass and Maurer, 2002). Ivermectin was found to be very highly bound to plasma protein (99.86%). Thus, an fu value of 0.0014 was applied in all simulations. The red blood cell/plasma cell partitioning of ivermectin has not been determined. Thus, a B:P ratio of 0.71 was predicted for ivermectin using algorithms within the Simcyp Simulator based on knowledge of log P, plasma fu, ionisation state (neutral) and haematocrit.

An *in vivo* V_ss_ value for ivermectin following IV administration was not available. Thus, a V_ss_ value was predicted using the following equation from Sawada *et al*. (1984):

 (Equation 3)

Where V is the fractional body volume (L/kg) of a tissue (t), erythrocyte (e), and plasma (p), E:P is the erythrocyte:plasma ratio and P_t:p_ is the partition coefficient for non-adipose and adipose components. Two methods are available for prediction of P_t:p_, the first reported by Poulin and Theil (2002) and modified by Berezhkovskiy (2004) and the second by Rodgers and Rowland (2005; 2006). The second method considers compound type (acid, base, neutral) as well as more extensive binding to intracellular tissue components. As ivermectin is neutral at physiological pH, only the first 2 methods were used to obtain estimates of V_ss_ for ivermectin and assessed in initial simulations. Predicted V_ss_ values were obtained using methods 1 (12.6 L/kg) and 2 (61.08 L/kg), for estimation of tissue partitioning (P_t:p_), including that of adipose, in Equation 3. Lipid partitioning is known to be higher for lipophilic unionised drugs, which is consistent with method 2 (Rodgers & Rowland, 2007).

While the steady-state volume of distribution in humans has not been determined, a healthy volunteer study has shown that the mean apparent volume of distribution (Vz/F) following oral administration of 12 mg ivermectin is 3.52 L/kg. Assuming that about 57% of the drug is absorbed (based on a 12 mg dose; Edwards *et al*., 1988), Vz is estimated to be about 2.02 L/kg. In a population PK model, central and peripheral volumes of distribution were estimated to be 1.27 and 3.34 L/kg, respectively (Duthaler *et al*., 2019). Although not directly comparable to the predicted V_ss_ values, these values do provide a reference point.

***In vitro metabolism***

Ivermectin is extensively metabolised in HLM via hydroxylation and demethylation (Zeng *et al*., 1998). Inhibition in HLM by both troleandomycin (87.17%) and an anti-human CYP3A4 antibody, indicated that ivermectin metabolism is mainly mediated by CYP3A4. This was confirmed in experiments with cells recombinantly expressing CYP1A1, 1A2, 2A6, 2B6, 2C8, 2C9, 2C19, 2D6, 2E1 or 3A4 (rCYP3A4), where only rCYP3A4 showed demonstrable turnover of ivermectin.

**Table S1.** Kinetic parameters for ivermectin in HLM

| Kinetic parameter | V_max_ | K_m_ |
| --- | --- | --- |
|  | nmol/min/mg | uM |
| HLM-JS | 0.61 | 32 |
| HLM-UC9504 | 0.49 | 42 |
| HLM-UC9411 | 0.23 | 48 |
| **Mean** | **0.44** | **40.67** |

The CL_H,int_ value of 10.9 µL/min/mg (0.44*1000/40.67) derived from the HLM data was then apportioned to CYP3A4 (87.2%) and other pathways (13.8%). In another study (Tipthara *et al*., 2021), 13 different metabolites (M1- M13) were identified after incubation of ivermectin with HLM. Three metabolites (M1: 3″-O-demethyl-ivermectin; M3: 4-hydroxymethyl-ivermectin; M6: 3″-O-demethyl, 4-hydroxymethyl-ivermectin) were amongst the major ones found in microsomes, hepatocytes, and blood from volunteers after oral ivermectin administration. *In vitro* metabolism studies indicated that M1, M3, and M6 were produced primarily by CYP3A4, and that M1 was also produced to a small extent by CYP3A5.

### *Retrograde model to determine all clearance routes*

A retrograde model was used within the Simcyp Simulator to estimate the hepatic intrinsic clearance (CLu_H,int_) from an oral clearance (CL/F – 15.8 L/h; Edwards *et al*., 1988) (fasted state) using Equation 4:

(Equation 4)

Where B:P is the concentration ratio of drug in blood to plasma (0.70); fu_b_ is fraction of unbound drug in blood (calculated from fu_p_/B:P; 0.0014/0.70); Q_H_ is the blood flow in the hepatic vein (76.5 L/hr); f_G_ is the fraction escaping first pass metabolism in the gut (assumed to be 1); CL_R_ is the renal clearance (assumed to be negligible); fa is the fraction absorbed (assumed to be 0.57 for the purposes of the calculation); Uptake is a factor that accounts for any active hepatic uptake (assumed to be the default value = 1). A value of 1638 µL/min/mg microsomal protein was estimated for CLu_H,int_ using Equation 4. The CL_H,int_ value (10.9 µL/min/mg) derived from *in vitro* data in its current form was not corrected for non-specific microsomal binding (NSMB). As this was not measured, an fu_mic_ value was predicted using a built-in algorithm based on log P (Turner *et al.,* 2006). This predicted parameter is highly sensitive to changes in log P (Table S2). After correcting the CL_H,int_ for fu_mic_, the resultant CLu_H,int_ was then expressed as a percentage of the metabolic clearance estimated from clinical data.

Ivermectin was identified as a P-gp substrate (Lespine *et al*., 2011) and appears to undergo significant biliary excretion via this transporter (Prichard *et al*., 2012). This is supported by the finding that accumulation of ivermectin (10-fold) occurred in the gall bladder cells in P-gp deficient mice relative to control mice (Schinkel *et al*., 1994). Furthermore, in 42 patients treated with ivermectin, a difference in MDR1 variant allele frequency was observed between suboptimal responders and responders (Kudzi *et al*., 2010). Thus, after subtracting the *in vitro* metabolism from the clinical component, the remaining was then assigned to biliary clearance.

The relative contribution of metabolism to the overall clearance of ivermectin, which was dependent on the log P value, ranged from 1.5 to 28.9% (Table 2). Assuming that a log P of 5.83 is the most appropriate value, the resultant CLu_H,int_ of 126.76 µL/min/mg protein was assigned to both CYP3A4 (110.5 µL/min/mg protein) and other pathways (16.27 µL/min/mg protein). The biliary clearance component of 1512 µL/min/mg protein was converted to units of µL/min/million cells (512=1512*39.79/117.51).

**Table S2.** Effect of log P on relative contribution of metabolism to the clearance of ivermectin

| CL_H,int_ | log P | Predicted | CLu_Hint_ | CLu_Hint_ | Biliary | % metabolism |
| --- | --- | --- | --- | --- | --- | --- |
| (*in vitro*) |  |  | (in vitro) | (clinical) |  |  |
| uL/min/mg |  | fu_mic_ | uL/min/mg | uL/min/mg | uL/min/mg |  |
| 10.90 | 4.10 | 0.44 | 25.00 | 1638 | 1613 | 1.53 |
| **10.90** | **5.83** | **0.09** | **126.76** | **1638** | **1512** | **7.74** |
| 10.90 | 7.00 | 0.02 | 473.98 | 1638 | 1164 | 28.93 |

###

### *Initial input parameters for ivermectin simulations*

All initial input parameters used for simulation of ivermectin kinetics during the model development stage are shown in Table S3.

**Table S3.** Input data for ivermectin

| **Parameter** | **Value** | **Source/Reference** |
| --- | --- | --- |
| MW | 875.1 |  |
| **cLog P** | **5.83 - >7** |  |
| Compound type | Neutral |  |
| **Aqueous solubility (mg/mL)** | **0.000175 – 0.004** | Takano *et al.* (2006); Rahman (2016) |
| fu | 0.0014 | Experimental |
| B:P | 0.71 | Predicted |
| V_ss_ (L/kg) | 61.08 | Predicted Method 2 |
| P_t:p_ adipose | 157 | Predicted Method 2 |
| CL_int,u_  (µl/min/mg protein) |  | Retrograde model was applied using CL_po_ of 15.8 L/h (Edwards *et al.*, 1988) (fasted) |
| CYP3A4  Other | 110.5  16.27 | Zeng *et al*. (1998) |
| Biliary | 1512 |  |
| Biliary (µl/min/million)* | 512 | Prichard *et al.* (2012) |

*This has been converted from a HLM to a hepatocyte input to reflect a biliary clearance i.e. multiplied by MPPGL and divided by HPGL. **Bold** indicates sensitive parameters with significant uncertainty.

***PART B:*** ***Model development for ivermectin***

### *Estimation of parameters with uncertainty – log P and solubility*

A full PBPK model in combination with the ADAM module was applied in preliminary simulations of plasma concentrations of ivermectin following oral administration of the drug. Using the inputs for ivermectin (Table S3), a sensitivity analysis was performed over the aqueous solubility range of 0.0001 to 0.004 mg/mL to determine the effect on fraction absorbed (fa) after oral administration of 12 mg ivermectin (Figure S1). This was repeated twice, initially considering aqueous solubility only (blue line) and then accounting for bile-micelle solubility using a partition coefficient predicted from a log P value of 5.83 (orange line). Both aqueous solubilities of 0.000128 mg/mL (combined with predicted bile micelle-mediated solubility) and 0.00152 mg/mL (no bile micelle-mediated solubility) led to a predicted fa of 0.57, which is consistent with the observed fa following administration of 12 mg in tablet form (Edwards *et al*., 1998).


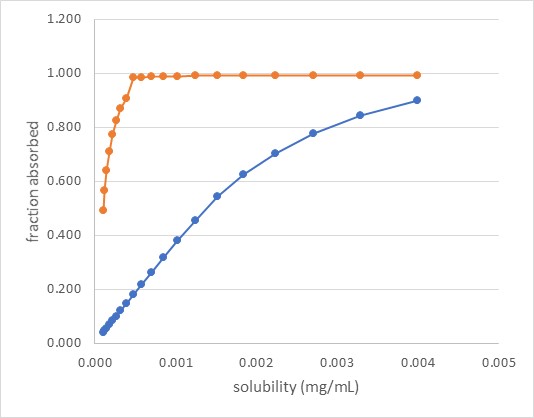


**Figure S1.** The effect of aqueous solubility on predicted fa

Using the inputs for ivermectin (Table S3), another sensitivity analysis was performed to look at the effect of dose on the fraction absorbed using an aqueous solubility of 0.000128 mg/mL (including bile micelles; orange) and 0.00152 mg/mL (excluding bile micelles; blue) (Figure S2). Both scenarios led to predicted fa values of 0.3 and 0.45 at the 30 mg dose, which would yield a significant food effect (observed is about 2.5-fold – Guzzo *et al*., 2002). However, it should be noted that based on the findings of Rahman (2016) and Takano *et al*. (2006), which indicate that bile micelle formation is significant for enhanced ivermectin solubility, an aqueous solubility of 0.000128 mg/mL (including bile micelles) was used going forward. The model could be quite sensitive to changes in factors that affect bile salt concentrations including food and diet.


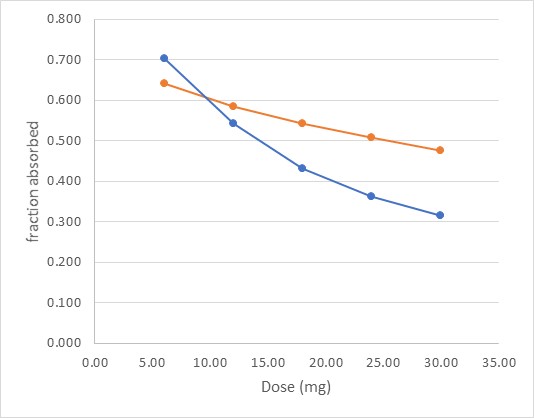


**Figure S2.** The effect of dose and solubility on the predicted fa for ivermectin

### *Comparison of simulated and observed plasma concentrations of ivermectin following a single oral dose of 12 mg – recovery of complex profile*

Ten virtual trials of 12 healthy male subjects aged 18 to 50 years receiving a single oral dose of 12 mg ivermectin in the fasted state were generated and the simulated (n=120) and observed (n=12; Edwards *et al*. (1998)) plasma concentrations and PK of ivermectin were compared. Using the inputs for ivermectin (Table S3) in a full PBPK model did not allow recovery of the observed profiles of ivermectin (data not shown). Based on the profiles, it appears that there is a much smaller volume of distribution initially followed by a much higher value as the drug partitions more extensively into adipose. Thus, the automated sensitivity analysis module was used to generate plasma concentration-time profiles using a range of values for a generic tissue scalar that was applied to all tissue P_t:p_ values (with the net effect of reducing the volume) and a range of values for the P_t;p_ describing the partitioning of the drug into adipose, that allowed improved recovery of the observed data from Edwards *et al.* (1988) (Figure S3). Final values of 0.1 and 5.75 were obtained for the generic tissue scalar and the adipose P_t:p_, respectively.

These final input values for ivermectin are as shown in Table S4 and were applied in all subsequent simulations.


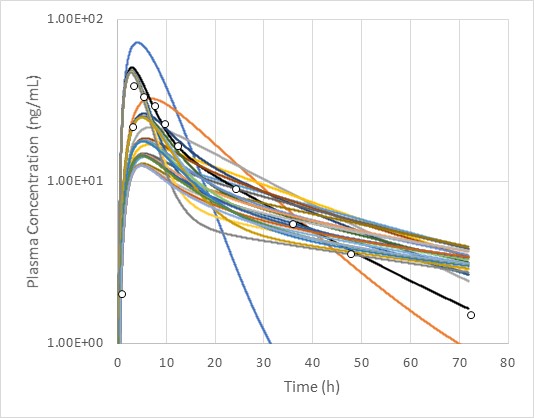


**Figure S3.** Log-linear plots of simulated *versus* observed (Edwards *et al*., 1988) ivermectin plasma concentration time profiles using a range of values for a generic tissue scalar and adipose P_t:p_.

| **Parameter** | **Value** | **Source/Reference** |
| --- | --- | --- |
| MW | 875.1 |  |
| cLog P | 5.83 |  |
| Compound type | Neutral |  |
| Aqueous solubility (mg/mL) | 0.000128 | Sensitivity analysis guided by clinical data (fraction absorbed) from Edwards *et al.* (1988) and solubility values from Takano *et al.* (2006) and Rahman (2016) |
| fu | 0.0014 |  |
| B:P | 0.71 | Predicted |
| V_ss_ (L/kg) | 2.14 | Predicted Method 2 |
| Tissue scalar | 0.1 | Optimised using profile from Edwards *et al.* (1988) |
| P_t:p_ adipose | 5.75 |  |
| CL_int,u_  (µl/min/mg protein) |  | Retrograde model was applied using CL_po_ of 15.8 L/h (Edwards *et al.*, 1988) (fasted) |
| CYP3A4  Other | 110.5  16.27 | Zeng *et al*. (1998) |
| Biliary | 1512 |  |
| Biliary (µl/min/million)* | 512 | Prichard *et al.* (2012) |

**Table S4.** Final input data for the ivermectin PBPK model

***PART C: Model verification for ivermectin***

## *Paediatric African population*

In a recent publication (Hayes *et al.*, 2015), a weight-for-age database was constructed from pre-existing population-based anthropometric data obtained from household surveys and research groups. It contained data collected between 1995 and 2012 on 1,263,119 individuals (909 368 female, 353 751 male), older than 14 days and younger than 50 years in 64 malaria-endemic countries. These growth charts were used to establish demographic profiles for the respective age groups used in the simulations. Simulations of 1000 children and adolescents aged 4 to <18 years were run. The weights of the individual virtual subjects are shown below for males and females (Figure 4S) with the WHO demographic profiles overlaid for both. These demographics were applied for all simulations involving African children.


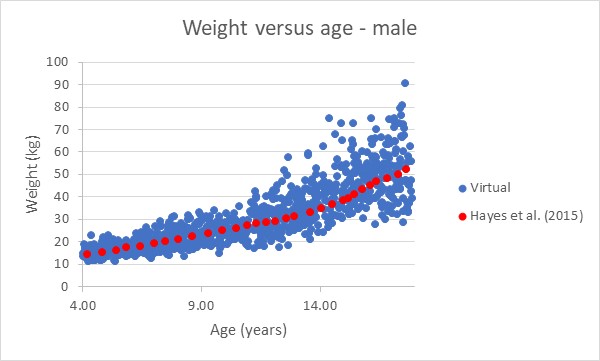

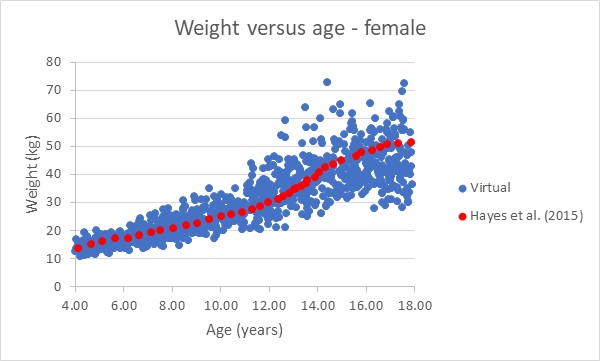


**Figure 4S.** Simulated (blue) *versus* observed (red) weights for male and female paediatric subjects aged 4 to 17 years.

### References

Badhan R, Zakaria Z, Olafuyi O. The Repurposing of Ivermectin for Malaria: A Prospective Pharmacokinetics-Based Virtual Clinical Trials Assessment of Dosing Regimen Options. *J Pharm Sci.* 2018 Aug;107(8):2236-2250. doi: 10.1016/j.xphs.2018.03.026. Epub 2018 Apr 5. PMID: 29626533.

Berezhkovskiy LM. Volume of distribution at steady state for a linear pharmacokinetic system with peripheral elimination. *J Pharm Sci*  2004; 93:1628-1640.

Duthaler U, Suenderhauf C, Karlsson MO, Hussner J, Meyer Zu Schwabedissen H, Krähenbühl S, Hammann F. Population pharmacokinetics of oral ivermectin in venous plasma and dried blood spots in healthy volunteers. Br J Clin Pharmacol. 2019 Mar;85(3):626-633. doi: 10.1111/bcp.13840. Epub 2019 Jan 24. PMID: 30566757; PMCID: PMC6379217.

Edwards G, Dingsdale A, Helsby N, Orme ML, Breckenridge AM. The relative systemic availability of ivermectin after administration as capsule, tablet, and oral solution. Eur J Clin Pharmacol. 1988;35(6):681-4. doi: 10.1007/BF00637608. PMID: 3234475.

Guzzo CA, Furtek CI, Porras AG, Chen C, Tipping R, Clineschmidt CM, Sciberras DG, Hsieh JY, Lasseter KC. Safety, tolerability, and pharmacokinetics of escalating high doses of ivermectin in healthy adult subjects. *J Clin Pharmacol*. 2002 Oct;42(10):1122-33. doi: 10.1177/009127002401382731. PMID: 12362927.

Hayes DJ, van Buuren S, ter Kuile FO, Stasinopoulos DM, Rigby RA, Terlouw DJ. Developing regional weight-for-age growth references for malaria-endemic countries to optimize age-based dosing of antimalarials. Bull World Health Organ. 2015 Feb 1;93(2):74-83.

Katneni K, Pham T, Saunders J, Chen G, Patil R, White KL, Abla N, Chiu FCK, Shackleford DM, Charman SA. Using Human Plasma as an Assay Medium in Caco-2 Studies Improves Mass Balance for Lipophilic Compounds. Pharm Res. 2018 Sep 17;35(11):210. doi: 10.1007/s11095-018-2493-3. PMID: 30225649; PMCID: PMC6156755.

Klotz U, Ogbuokiri JE, Okonkwo PO. Ivermectin binds avidly to plasma proteins. Eur J Clin Pharmacol. 1990;39(6):607-8. doi: 10.1007/BF00316107. PMID: 2095348.

Kudzi W, Dodoo AN, Mills JJ. Genetic polymorphisms in MDR1, CYP3A4 and CYP3A5 genes in a Ghanaian population: a plausible explanation for altered metabolism of ivermectin in humans? *BMC Med Genet.* 2010 Jul 14;11:111. doi: 10.1186/1471-2350-11-111. PMID: 20630055; PMCID: PMC3161347.

Lespine A, Ménez C, Bourguinat C, Prichard RK. P-glycoproteins and other multidrug resistance transporters in the pharmacology of anthelmintics: Prospects for reversing transport-dependent anthelmintic resistance. *Int J Parasitol Drugs Drug Resist.* 2011 Nov 7;2:58-75. doi: 10.1016/j.ijpddr.2011.10.001. PMID: 24533264; PMCID: PMC3862436.

Poulin P and Theil FP. Prediction of pharmacokinetics prior to in vivo studies 1. Mechanism-based prediction of volume of distribution. *J Pharm Sci* 2002; 91:129-156.

Prichard R, Ménez C, Lespine A. Moxidectin and the avermectins: Consanguinity but not identity. *Int J Parasitol Drugs Drug Resist*. 2012 Apr 14;2:134-53. doi: 10.1016/j.ijpddr.2012.04.001. PMID: 24533275; PMCID: PMC3862425.

Rahman S (2016). Analysis of surfactant-mediated dissolution effects in biorelevant media. 2016. PhD. University of Maryland, Baltimore.

Rodgers T, Leahy D and Rowland M. Physiologically based pharmacokinetic modeling 1: predicting the tissue distribution of moderate-to-strong bases. *J Pharm Sci* 2005; 94:1259-1276.

Rodgers T and Rowland M. [Physiologically based pharmacokinetic modelling 2: predicting the tissue distribution of acids, very weak bases, neutrals and zwitterions.](http://www.ncbi.nlm.nih.gov/pubmed/16639716?ordinalpos=6&itool=EntrezSystem2.PEntrez.Pubmed.Pubmed_ResultsPanel.Pubmed_DefaultReportPanel.Pubmed_RVDocSum) *J Pharm Sci.* 2006; 95(6):1238-57.

Rodgers T, Rowland M. Mechanistic approaches to volume of distribution predictions: understanding the processes. Pharm Res. 2007 May;24(5):918-33. doi: 10.1007/s11095-006-9210-3. Epub 2007 Mar 20. PMID: 17372687.

Sawada Y, Hanano M, Sugiyama Y, Harashima H and Iga T. Prediction of the volumes of distribution of basic drugs in humans based on data from animals. *J Pharmacokinet Biopharm* 1984; 12:587-596.

Schinkel AH, Smit JJ, van Tellingen O, Beijnen JH, Wagenaar E, van Deemter L, Mol CA, van der Valk MA, Robanus-Maandag EC, te Riele HP, et al. Disruption of the mouse mdr1a P-glycoprotein gene leads to a deficiency in the blood-brain barrier and to increased sensitivity to drugs. *Cell.* 1994 May 20;77(4):491-502. doi: 10.1016/0092-8674(94)90212-7. PMID: 7910522.

Sugano K. (2009a) Estimation of effective intestinal membrane permeability considering bile micelle solubilisation. *Int J Pharm* 2009a; 368:116-122.

Sugano K (2009b) Theoretical investigation of passive intestinal membrane permeability using Monte Carlo method to generate drug-like molecule population. *Int J Pharm* 2009b; 373:55-61.

Takano R, Sugano K, Higashida A, Hayashi Y, Machida M, Aso Y, Yamashita S. Oral absorption of poorly water-soluble drugs: computer simulation of fraction absorbed in humans from a miniscale dissolution test. *Pharm Res.* 2006 Jun;23(6):1144-56. doi: 10.1007/s11095-006-0162-4. Epub 2006 May 25. PMID: 16715363.

Tipthara P, Kobylinski KC, Godejohann M, Hanboonkunupakarn B, Roth A, Adams JH, White NJ, Jittamala P, Day NPJ, Tarning J. Identification of the metabolites of ivermectin in humans. Pharmacol Res Perspect. 2021 Feb;9(1):e00712. doi: 10.1002/prp2.712. PMID: 33497030; PMCID: PMC7836931.

Turner DB, Rostami-Hodjegan A, Tucker GT and Rowland-Yeo K. Prediction of non-specific microsomal binding from readily available physicochemical properties. *9^th^ European ISSX Meeting, Manchester, UK, June 4-7, 2006.*

World Health Organization. Growth reference 5-19 years;Available from: http:// www.who.int/growthref/en/.

Zeng Z, Andrew NW, Arison BH, Luffer-Atlas D, Wang RW. Identification of cytochrome P4503A4 as the major enzyme responsible for the metabolism of ivermectin by human liver microsomes. Xenobiotica. 1998 Mar;28(3):313-21. doi: 10.1080/004982598239597. PMID: 9574819.
